# Prognostic Significance of Percutaneous Coronary Intervention for First Acute Myocardial Infarction with Heart Failure: Five-Year Follow-Up Results

**DOI:** 10.1155/2022/5791295

**Published:** 2022-11-03

**Authors:** Zichao Cheng, Yuchen Shi, Hongyu Peng, Donghui Zhao, Qian Fan, Jinghua Liu

**Affiliations:** Center for Coronary Artery Disease (CCAD), Department of Cardiology, Beijing Anzhen Hospital, Capital Medical University,and Beijing Institute of Heart,Lung and Blood Vessel Diseases, Beijing, China

## Abstract

**Objective:**

The study aimed to investigate the incidence and influencing factors of heart failure after 5 years of percutaneous coronary intervention (PCI) for first acute myocardial infarction.

**Methods:**

A total of 1235 patients, diagnosed as acute myocardial infarction and treated with PCI in Beijing Anzhen Hospital, Capital Medical University, from January 1, 2014, to December 31, 2014, were enrolled. Based on the exclusion criteria, 671 patients were followed up to obtain echocardiographic results 5 years after the onset of myocardial infarction (from January 1, 2019, to December 31, 2019). Of 671 patients, 62 were lost to follow-up. Finally, 609 patients were recruited in this study. According to the results of the echocardiographic examination, patients were divided into a heart failure group (*n* = 97) (LVEF < 50%) and a nonheart failure group (*n* = 512) (LVEF ≥ 50%). The clinical characteristics were compared between the two groups, and the influencing factors of heart failure after 5 years of PCI in patients with acute myocardial infarction were analyzed using logistic regression and receiver-operating characteristic (ROC) analyses.

**Results:**

Of 609 patients, 97 had heart failure within 5 years after PCI for first myocardial infarction, with an incidence of 15.9%. Multivariate regression analysis finally examined the predictors related to the occurrence of heart failure, including age (aOR, 1.008; 95% confidence interval (CI), 1.054–1.123; *P* ≤ 0.001), peak troponin I level (aOR, 1.020; 95% CI, 1.006–1.034; *P* = 0.004), left ventricular ejection fraction (LVEF) (during admission) (aOR, 0.908; 95% CI, 0.862–0.956; *P* ≤ 0.001), and left ventricular end-diastolic dimension (LVEDD) (at admission) (aOR, 1.136; 95% CI, 1.016–1.271; *P* = 0.025).

**Conclusion:**

In this study, the incidence of heart failure (LVEF < 50%) in patients with acute myocardial infarction who underwent PCI was 15.9% at a five-year follow up. Age, peak troponin I level, and LVEDD (at admission) were risk factors for heart failure, while LVEF (at admission) of patients during hospitalization was a protective factor for heart failure.

## 1. Introduction

Coronary artery disease (CAD), which is approximately responsible for 9 million deaths in 2016, is one of the leading causes of death globally [1]. Acute myocardial infarction (AMI) is one of the most serious manifestations of CAD. The increasing use of coronary revascularization and advancement in medical therapy have improved survival rates of patients with AMI [2].^.^ A large-scale epidemiological survey shows that in China, the in-hospital mortality rate of AMI is approximately 6.58% [3], while most patients have long-term survival after having AMI. Some patients continue to develop heart failure (HF) for several years after percutaneous coronary intervention (PCI), resulting in repeated hospitalizations and even death. It has been proven that the improved treatment of AMI has contributed to the epidemic of HF [4–7].

The results of a prospective study in the United Kingdom in 12 years (1998–2010) suggest that approximately one in four patients will develop HF within 4 years of first AMI [8]. A Swedish study found that the cumulative risk of HF 5 years after AMI was 21.8% [9]. These studies indicate that the incidence of HF after AMI is high. It is necessary to detect and identify patients with AMI who are at risk of progression to HF and provide adequate and even preventive HF treatment as soon as possible.

Relevant studies have identified risk factors for HF after AMI, such as old age, severity of CAD, infarct size, and adequacy of reperfusion therapy [10, 11]. However, there are still insufficient studies on patients with first AMI. The prediction of HF after AMI by clinicians is still subjective and empirical. Therefore, the control rate of HF after AMI is still low. In an electronic health record cohort study in England (1998–2010), only 9.2% of patients underwent primary PCI [8]. Moreover, previous studies on HF after AMI reported that it is difficult to ensure that patients receive a timely and uniform rescue process [8, 9, 12], which is greatly important in the treatment of patients with AMI.

Therefore, this retrospective study aimed to determine the incidence and identify independent predictors of HF after 5 years of PCI of first AMI. In this study, all patients with first AMI received PCI in our center, which is one of the largest heart centers in China.

## 2. Methods

### 2.1. Study Population

This study, following the 2013 Declaration of Helsinki [13], was approved by the institutional review board and deemed exempt from informed consent requirements.

**Figure 1:**
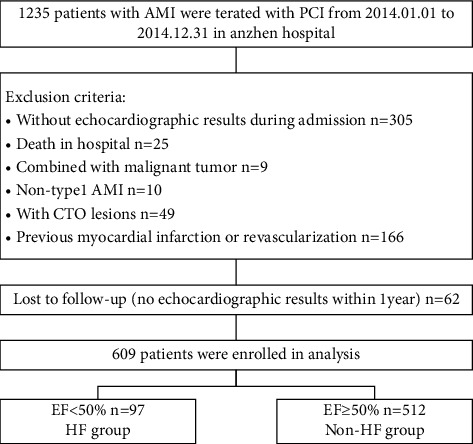
Flowchart of this study.

All patients were identified from a retrospective review of the institution's database with records of detailed information, including baseline characteristics, laboratory examinations, echocardiographic measurements, in-hospital angiographic characteristics, and medications used during hospitalization. From January 1, 2014, to December 31, 2014, 1235 patients diagnosed with AMI including ST-segment elevation myocardial infarction (STEMI) and non-ST segment elevation myocardial infarction (NSTEMI) were treated with PCI in AnZhen Hospital. According to the exclusion criteria, 671 patients were included (Figure 1.). These patients were followed up with echocardiography (from January to December 2019) in our hospital to determine cardiac function. During the follow-up, 62 patients were lost. Finally, a total of 609 patients were enrolled in this study. Figure 1 shows the flowchart of the enrolled population in this study.

### 2.2. Data Collection

The clinical data recorded in this study included demographic characteristics, laboratory examination during hospitalization, echocardiographic results, details of PCI treatment during hospitalization, and medication regimen. The demographic data included age, sex, body mass index, and medical history, including smoking, hypertension, diabetes, atrial fibrillation, and stroke. Laboratory examination included peak troponin I (TNI) level, peak creatine kinase-MB (CK-MB), white blood cell (WBC) count, neutrophil count ratio, platelet count, hemoglobin count, high-sensitivity C-reactive protein (Hs-CRP), uric acid, creatinine, cereal third transaminase (ALT), aspartate aminotransferase (AST), total protein, total bilirubin, urea nitrogen, HbA1c, fasting plasma glucose, triglyceride, high-density lipoprotein cholesterol, low-density lipoprotein cholesterol, hematocrit, and brain natriuretic peptide (BNP) levels. Echocardiographic results during admission, including left ventricular ejection fraction (LVEF), left ventricular end-diastolic dimensions (LVEDD), left ventricular end-systolic dimension (LVESD), segmental wall motion abnormality, and ventricular aneurysm, were also recorded. All examinations were performed within 24 h of admission. In-hospital angiographic characteristics included AMI type (STEMI/NSTEMI), preinfarction angina, time from attack to PCI (h), thrombolytic therapy, PCI vessels, culprit vessels, single-vessel disease, TIMI flow (before and after PCI), absence of reflow, complete revascularization, and use of the intra-aortic balloon pump (IABP) and extracorporeal membrane oxygenation (ECMO). Medication use during hospitalization was also included.

### 2.3. Statistical Analyses

The data were analyzed using the SPSS statistical package, version 22.0 (IBM corporation, Armonk, NY). Continuous variables were presented as a mean ± standard deviation or median (range) values and compared using the independent-sample *t*-test (data with normal distribution) or the Mann–Whitney *U* test (data with non-normal distribution). Categorical variables were expressed as numbers (percentage) of patients in each group and analyzed using the chi-squared test. Variables with a statistical significance between the two groups were incorporated into a single-factor logistic regression analysis to select meaningful predictors and entered into the multifactor logistic regression analysis to finally obtain independent predictors of HF. The ROC curve based on the logistic model was established to evaluate the prediction probability. A *P* value <0.05 (bilateral) was statistically significant.

### 2.4. Definition

AMI caused by atherothrombotic CAD and usually precipitated by atherosclerotic plaque disruption (rupture or erosion) is designated as type 1 AMI [14].

Chronic total occlusion (CTO) is defined as a coronary lesion with TIMI flow grade 0 of at least three months duration, which is frequently encountered during coronary angiography in patients with coronary artery disease (CAD) [15]. The definition of complete revascularization is as follows: noninfarcted vessels with significant stenosis (diameter stenosis rate 70%) were also treated during PCI [16]. HF is defined as an ejection fraction (EF) < 50%. Previous AMI was defined as a clear history of NSTEMI or STEMI or a Q-wave on electrocardiography. Previous revascularization is defined as previous PCI or coronary artery bypass grafting or thrombolytic therapy for AMI.

Preinfarction angina was defined as ≥1 episode of angina within 48 h prior to AMI [17]. Culprit vessels of STEMI are determined by the characteristic ST-segment elevation or *Q*-wave formation on electrocardiography. Culprit vessels of NSTEMI were determined by an experienced cardiologist based on angiographic results, electrocardiographic changes, and echocardiographic findings.

The definition of HF is EF < 50% with presence of symptoms and/or signs. This definition includes HFrEF (EF ≤ 40%) and HFmrEF (41% < EF ≤ 49%) [18].

## 3. Results

### 3.1. Incidence of HF

A total of 671 patients met the inclusion criteria. During the follow-up, 62 patients were lost. Finally, 609 patients were included in the statistical analysis. Based on the results of the patient's echocardiography in 2019, 97 patients in the HF group (LVEF < 50%) and 512 patients in the non-HF group (LVEF ≥ 50%) were analyzed. Figure 1 shows the flowchart of the enrolled population in this study.

### 3.2. Baseline Characteristics, Laboratory Examinations, and Echocardiographic Measurements

Table 1 shows baseline data, laboratory examination, and echocardiographic results of patients in the two groups. In terms of baseline characteristics, patients in the HF group were older and had lower systolic blood pressure than patients in the non-HF group. Regarding laboratory examinations, many examination results of patients in the HF group were higher than those in the non-HF group, including WBC count, neutrophil count, hematocrit, TNI, CK-MB, Hs-CRP, ALT, total bilirubin, fasting plasma glucose, and BNP levels.

### 3.3. In-Hospital Angiographic Characteristics

Furthermore, the results of the comparison of angiography and reperfusion therapy during hospitalization between the two groups of patients were as follows (Table 2): first, the proportion of patients with STEMI in the HF group was significantly higher than that in the non-HF group, and the proportion of patients with preinfarction angina in the HF group was significantly lower than that in the non-HF group. However, there was no significant difference in the time from attack to PCI between the two groups. In terms of target vessels of PCI, patients in the HF group received significantly higher percentage of the LAD artery and RCA revascularization than those in the non-HF group, but no significant difference was found in the LCX. The same results were obtained in terms of culprit vessels. In terms of vascular disease severity, single-vessel disease was more likely to develop in patients with HF than in patients without HF. As for TIMI blood flow before PCI, the HF group tended to have more TIMI grade flow 0, while the non-HF group tended to have more TIMI grade flow 3. There was no difference in TIMI flow after PCI between the two groups. There were no differences in absence of reflow, stent placement, and complete revascularization between the two groups.

### 3.4. Medication

Table 3 compares the difference in drug use during hospitalization between patients in the HF group and those in the non-HF group. It was found that all patients used aspirin and either clopidogrel or ticagrelor. There was no significant difference in the antiplatelet agent and low-molecular-weight heparin (LMWH) use; however, tirofiban use was significantly higher in the HF group than in the non-HF group. Use of other drugs, beta-blockers and spironolactone, was significantly different between the two groups, and patients in the HF group tended to use more of both drugs. The abovementioned differences were statistically significant.

### 3.5. Logistic Regression Analysis and ROC Curve Analysis

**Figure 2:**
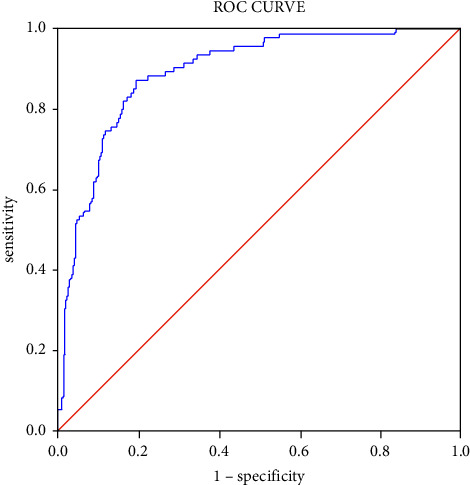
ROC curve analysis.

Logistic regression analysis was performed on variables with significant differences between the two groups, and the results are shown in Table 4. In the univariate logistic regression analysis, age, WBC count, neutrophil count, TNI, CK-MB, Hs-CRP, AST, total bilirubin, fasting blood glucose levels, LVEDD, LVESD, STEMI, preinfarction angina, LAD (target) artery, RCA (target), LAD (culprit) artery, RCA (culprit), single lesion, TIMI-0 (before PCI), TIMI-3 (before PCI), and use of tirofiban and spironolactone were predictors of HF development. The results of the multivariate regression analysis showed that age (aOR 1.008; 95% confidence interval (CI), 1.054–1.123; *P* ≤ 0.001), peak TNI level (aOR, 1.020; 95% CI, 1.006–1.034; *P* = 0.004), and LVEDD (aOR, 1.136; 95% CI, 1.016–1.271; *P* = 0.025) were independent predictors of HF development, while LVEF (aOR, 0.908; 95% CI, 0.862–0.956; *P* ≤ 0.001) was a protective factor of HF development. As shown in Figure 2, the area of the logistic regression equation (*C*-index) was 0.896 (95% CI, 0.864–0.928; *P* ≤ 0.001).

## 4. Discussion

With the rapid development of revascularization technology and drug therapy, the main burden in AMI has changed from high in-hospital mortality to a poor prognosis after discharge [4]. Several studies have shown that approximately a quarter of patients develop HF within a few years of hospital discharge [8, 9, 19]. Predicting the risk of HF after AMI is of great significance in the prognosis and treatment of AMI.

Compared with many previous studies [8, 9, 19], a novel definition of heart failure (EF < 50%) was used in this study. Traditionally, HF has been divided into distinct phenotypes based on the measurement of the left ventricular ejection fraction (LVEF) [18]. The rationale behind this relates to the original treatment trials in HF that demonstrated substantially improved outcomes in patients with an LVEF < 40% [18]. However, HF spans the entire range of the LVEF (a normally distributed variable), and measurement by echocardiography is subject to substantial variability [18]. Compared with >40% in many previous studies [8, 9, 19], the definition of >50% can include more patients. The patients with HFpEF (those with symptoms and signs of HF, with evidence of structural and/or functional cardiac abnormalities and/or raised natriuretic peptides (NPs), and with an LVEF > 50%) were excluded in this study because its diagnosis is more complicated than the first two types of HF, and it is not solely dependent on EF examination. The occurrence of HFpEF in patients with AMI is a complicated process [12]. It involves the diastolic function of the heart, and there may be other specific pathological mechanisms [20, 21]. The incidence of HF in the study was 15.8% 5 years after first AMI, which was lower than those in previous studies when the diagnostic criteria for HF were more extensive [8]. The possible reason was that all patients in this study had first AMI and received PCI. This is also related to the higher level of treatment received by patients.

In this study, patients in the HF group were older, and there are sufficient data to show that advanced age can predict the risk of in-hospital death in patients with AMI [22]. Regarding the relationship between senile HF and AMI, studies have shown that aging may be a cause of HF after ischemia [23, 24]. Epidemiological studies have shown that in Europe and America, for 25 years, the number of patients with chronic HF increased by 70–100%. The reason for this may be that most patients with incipient AMI who received ischemia reperfusion therapy developed chronic HF years later [23, 24]. Basic studies have shown that senescence has the effect of inducing apoptosis of cardiomyocytes, which may be the mechanism of late-onset ischemic HF in elderly patients [25, 26]. The results of the multivariate regression analysis in this study showed that age was an independent predictor of HF (OR, 1.008; 95% CI, 1.054–1.123; *P* ≤ 0.001).

The infarction area in patients with AMI is closely related to death and long-term prognosis of patients during hospitalization. Since the myocardial size is difficult to regenerate, damaged cells are often replaced by cells without systolic function, so the infarction area can be an important factor in the development of HF. However, due to the high cost and complexity of accurate measurement of the AMI area, such as myocardial nuclide imaging and myocardial magnetic resonance imaging, such tests are often not used in the clinical treatment of patients with AMI. However, a variety of laboratory tests can indirectly or directly reflect the size of the infarction area in patients. Some studies have shown that the WBC count, neutrophil count, and CRP level are related to the inflammatory response caused by the expansion of the infarction area [27–30]. In this study, the WBC count, neutrophil count, and CRP level of patients in the HF group were higher than those in the non-HF group, but regression analysis failed to show that these indicators were independent predictors of HF, which may be related to the inability of these test results to accurately evaluate the infarction area.

Moreover, myocardial necrosis indicators (CK-MB and TNI) and liver biomarkers (TB, ALT, and AST) can also reflect the area size of myocardial injury. Studies have already confirmed that all myocardial enzyme levels are associated with adverse outcomes of AMI [31]. In this study, all myocardial enzyme levels of patients in the HF group were higher than those of patients in the non-HF group. Multiple factor regression analysis showed that the TNI level is an independent predictor of HF in patients with AMI, and the ROC curve analysis showed that TNI had the best sensitivity and specificity.

Others may be associated with the prognosis of AMI. However, the laboratory tests failed to predict the effect of HF after AMI occurrence. For example, a study reported that diabetes and stress hyperglycemia are the criteria of AMI in patients at high risk [32]. In this study, although blood glucose levels were higher in patients with HF, the predictive effect regression analysis did not find a significant difference. This may be because the hospital once measured blood glucose levels associated with the patient's stress and diet and did not reflect the true blood glucose level in patients [32]. Additionally, statistically significant differences were not found in various lipid tests between groups. Although studies have found that the renal function level in patients with AMI is an independent predictor of the prognosis of patients with AMI [33], this study failed to find a correlation between abnormal renal function and HF in the majority of patients with normal renal function. The abovementioned laboratory examination results need to be clarified by prospective studies because of the large uncontrollable factors in observational studies.

Liu et al. showed that the level of cardiac function during hospitalization in patients with AMI could predict adverse cardiac events after PCI, suggesting that decreased left ventricular diastolic function and left ventricular dilatation were predictors of adverse events after PCI [34]. In this study, patients in the HF group showed a significant difference in LVEF and LVEDD compared with those in the non-HF group, and regression analysis showed that LVEDD was an independent predictor of HF, while LVEF was a protective factor. The results showed that in patients with severely affected cardiac function after AMI, the risk of development of HF after AMI has increased significantly. On the one hand, cardiac function may reflect the severity of AMI. On the other hand, in the event of HF, a series of HF neurohumoral factors is activated (e.g., activation of the sympathetic nervous system and renin-angiotensin-aldosterone system). These factors may persist after discharge from the hospital. In this study, BNP levels in the HF group were higher than those in the non-HF group, but the predictive effect of BNP was not found in the regression analysis. This may be due to the difficulty in controlling the influential factors of laboratory examination results in observational studies, which need to be further clarified by prospective studies. Regarding ventricular wall motion abnormality and ventricular wall tumors, some studies have found that the score of ventricular wall motion abnormality based on the echocardiographic results is of great significance in the determination of AMI severity. However, because its determination is relatively subjective, no significant difference was found between the two groups in this study. Similarly, there was no significant difference between the HF and non-HF groups. For such indicators, further research is needed to evaluate them on the premise of accurate detection.

It has been reported that patients with angina pectoris before infarction who received thrombolytic therapy had significantly lower rates of cardiogenic shock, malignant arrhythmia, and mortality than patients without angina pectoris before infarction [35]. More data indicate that preinfarction angina pectoris has a myocardial protective effect on AMI, and its mechanism may be related to myocardial ischemic preconditioning induced by preinfarction angina pectoris [35]. In this study, the incidence of preinfarction angina in the HF group was significantly lower than that in the non-HF group, confirming the results of the abovementioned study.

The results of this study showed that the type of AMI was not a predictor of HF after AMI, although the HF group had a higher proportion of STEMI than the non-HF group, and despite differences between the two groups, neither the offender nor the target vessel was a predictor of HF. The results are consistent with those of a large study in the UK [8]. The relationship between the type of AMI and prognosis of AMI has long been controversial [8]. A study in Japan showed that patients with NSTEMI had poorer long-term prognosis than patients with STEMI and had a higher rate of HF after AMI [36]. This problem still needs to be further elaborated by prospective studies.

As discussed above, the incidence of HF (LVEF < 50%) in patients with AMI 5 years after PCI was 15.8%, among which age, peak TNI level, and LVEDD were risk factors for HF, while LVEF of patients during hospitalization was a protective factor for HF. In clinical work, patients with AMI with high-risk factors should be paid close attention. In these patients, the changes in cardiac function should be considered, and preventive and anti-HF treatment should be performed, when necessary. We have designed prospective studies to further clarify the findings of this study and will discuss in further work whether intervention with these risk factors can reduce the risk of HF in patients with AMI.

## 5. Limitations

There are some limitations in this study. This was a retrospective observational study that screened a subset of patients, which may have led to population selection bias. In addition, this study failed to distinguish the types of myocardial infarction, and different types may have different mechanisms. Finally, the treatment received by patients after discharge was not followed up accurately, which may affect patient outcomes. We will further design prospective studies and closely follow patients to further explore this issue.

## Figures and Tables

**Table 1:** Baseline characteristics, laboratory examinations, and echocardiographic measurements.

Variables	HF group (*n* = 97)	Non-HF group (*n* = 512)	*P*
Age (*Y*)	59.30 ± 11.56	52.07 ± 10.93	≤0.001
Sex male *n* (%)	79 (81.44)	419 (83.46)	0.972
Sex female *n* (%)	18 (18.55)	83 (16.53)	0.972
BMI (kg/m^2^)	25.94 ± 2.38	25.88 ± 2.44	0.328
Systolic pressure (mmHg)	117.98 ± 16.58	121.41 ± 16.63	0.003
Diastolic pressure (mmHg)	74.51 ± 11.44	73.83 ± 10.80	0.667
Smoking *n* (%)	61 (62.88)	314 (62.54)	0.682
Hypertension *n* (%)	39 (40.20)	263 (52.39)	0.053
Diabetes *n* (%)	25 (25.77)	142 (28.28)	0.725
Atrial fibrillation *n* (%)	3 (3.1)	6 (0.2)	0.151
Stroke *n* (%)	3 (3.1)	13 (2.6)	0.755

Laboratory examinations			
Peak troponin I (ng/mL)	54.00 ± 45.87	22.47 ± 30.47	≤0.001
Peak CKMB (ng/mL)	161.45 ± 133.48	89.69 ± 114.70	≤0.001
White blood cell count (10^9^/L)	10.06 ± 3.65	8.64 ± 2.86	0.002
Neutrophil count (10^9^/L)	7.58 ± 3.54	6.42 ± 2.74	≤0.001
Platelet count (10^9^/L)	218.75 ± 60.05	207.24 ± 56.17	0.237
Hemoglobin (g/L)	136.74 ± 21.13	140.61 ± 16.78	0.151
Hs-CRP (mg/L)	16.28 ± 13.62	11.16 ± 11.80	≤0.001
Uric acid (*μ*mol/L]	328.34 ± 80.07	334.12 ± 95.29	0.592
Creatinine (*μ*mol/L)	72.94 ± 19.24	75.43 ± 19.32	0.066
Homocysteine (*μ*mol/L)	17.35 ± 8.96	17.64 ± 9.65	0.720
ALT (U/L)	56.35 ± 33.79	41.95 ± 30.42	0.005
AST (U/L)	178.25 ± 164.835	99.32 ± 114.195	0.029
Total protein (g/L)	67.08 ± 3.97	66.36 ± 3.39	0.666
Total bilirubin (*μ*mol/L)	10.71 ± 6.17	9.18 ± 5.93	0.003
Urea nitrogen (mmol/L)	3.49 ± 1.86	3.22 ± 1.59	0.490
Hba1c (%)	6.51 ± 1.29	6.42 ± 1.28	0.305
Fasting plasma glucose (mmol/L)	13.66 ± 5.21	12.16 ± 4.01	0.012
Triglycerides (mmol/L)	3.05 ± 1.91	2.77 ± 1.90	0.539
HDL-C (mmol/L)	1.82 ± 0.73	1.89 ± 0.69	0.075
LDL-C (mmol/L)	2.04 ± 0.99	2.03 ± 1.01	0.953
Hematocrit (%)	62.08 ± 44.18	52.85 ± 54.78	0.008
BNP (pg/ml)	298.15 ± 260.99	230.60 ± 294.51	0.003

Echocardiographic measurements			
LVEF (%)	47.34 ± 8.64	56.49 ± 7.33	≤0.001
LVEDD (mm)	52.81 ± 5.75	48.70 ± 5.11	≤0.001
LVESD (mm)	38.81 ± 6.02	33.45 ± 5.52	≤0.001
Segmental wall movement abnormal	88 (90.72)	327 (64.49)	≤0.001
Ventricular aneurysm	10 (10.87)	15 (3.0)	0.019

BMI: body mass index; Hs-CRP: hypersensitive C-reactive protein; AST: aspartate aminotransferase; ALT: alanine aminotransferase; HDL-C: high-density lipoprotein cholesterol; LDL-C: low-density lipoprotein cholesterol; BNP: B-type natriuretic peptide; LVEF: left ventricular ejection fraction; LVEDD: left ventricular end-diastolic diameter; LVESD: left ventricular end systolic diameter.

**Table 2:** In-hospital angiographic characteristics.

Variables	HF group (*n* = 97)	Non-HF group (*n* = 512)	*P*
STEMI *n* (%)	84 (86.6)	359 (71.51)	≤0.001

Preinfarction angina *n* (%)	37 (38.14)	253 (50.4)	0.046

Length from attack to PCI(h)	56.44 ± 117.35	55.53 ± 87.47	0.069

Thrombolytic therapy *n* (%)	6 (6.18)	37 (7.37)	0.849

PCI vessels			
LMCA *n* (%)	0 (0)	3 (0.2)	0.450
LAD *n* (%)	65 (67.01)	265 (51.75)	0.003
LCX *n* (%)	21 (1.03)	144 (28.12)	0.210
RCA *n* (%)	24 (14.74)	201 (39.25)	0.026

Culprit vessels			
LAD *n* (%)	62 (63.91)	242 (47.26)	0.002
LCX *n* (%)	19 (19.58)	109 (21.28)	0.699
RCA *n* (%)	21 (21.65)	184 (35.93)	0.007

Single-vessel disease *n* (%)	53 (54.64)	226 (44.14)	0.039

TIMI flow			
TIMI flow before PCI			
0 *n* (%)	67 (69.07)	227 (44.33)	≤0.001
1*n* (%)	1 (1.03)	3 (0.58)	0.609
2*n* (%)	1 (1.03)	9 (1.75)	0.616
3*n* (%)	16 (16.49)	152 (29.69)	≤0.001
TIMI flow after PCI			
0–2*n* (%)	1 (1.08)	2 (0.39)	0.408
3*n* (%)	97 (100)	509 (99.41)	0.450

No reflow	3 (3.09)	14 (2.73)	0.844
Stent placement *n* (%)	57 (61.95)	292 (57.03)	0.215
Complete revascularization *n* (%)	34 (35.05)	182 (35.54)	0.561
IABP *n* (%)	3 (3.26)	1 (0.19)	0.053
ECMO *n* (%)	0	0	

STEMI: ST-segment elevation myocardial infarction; LMCA: left main coronary artery; LAD: left anterior descending artery; LCX: left circumflex artery; RCA: right coronary artery; IABP: intra-aortic balloon pump; ECMO: extracorporeal membrane oxygenation.

**Table 3:** Medication during hospitalization.

Variables	HF group (*n* = 97)	Non-HF group (*n* = 512)	*P*
Aspirin *n* (%)	57 (100)	301 (100)	
Clopidogrel *n* (%)	88 (90.72)	440 (85.93)	0.277
Ticagrelor *n* (%)	9 (9.27)	72 (14.06)	0.218
LMWH *n* (%)	95 (97.93)	498 (97.26)	0.704
GP IIb/IIIa inhibitor *n* (%)	61 (62.88)	258 (50.39)	0.040
*β*-Receptor blocker *n* (%)	92 (94.84)	451 (88.08)	0.050
ACEI/ARB *n* (%)	84 (86.59)	408 (79.68)	0.272
Loop diuretics *n* (%)	10 (10.30)	30 (5.85)	0.246
Statins *n* (%)	97 (100)	511 (99.80)	0.663
Spironolactone *n* (%)	11 (11.34)	14 (2.73)	≤0.001
Antidiabetic drugs *n* (%)	12 (12.37)	85 (16.60)	0.296
Insulin *n* (%)	10 (10.30)	44 (8.59)	0.5.86

LMWH: low-molecular-heparin; ACEI: angiotensin-converting enzyme inhibitors; ARB: angiotensin receptor blocker.

**Table 4:** Univariate and multivariate logistic regression analyses.

Variables	Univariate logistic regression			Multivariate logistic regression		
	OR	95% CI	*P*	OR	95% CI	*P*
Age	1.063	1.014–1.087	≤0.001	1.008	1.054–1.123	≤0.001
Systolic pressure	0.998	0.975–1.001	0.640			
Peak troponin I	1.002	1.016–1.028	≤0.001	1.020	1.006–1.034	0.004
Peak CK-MB	1.004	1.003–1.006	≤0.001	1.000	0.995–1.004	0.831
White blood cell count	1.128	1.056–1.206	≤0.001	0.921	0.614–1.384	0.693
Platelet count	1.005	1.002–1.009	0.202			
Neutrophil count	1.147	1.073–1.227	≤0.001	1.055	0.693–1.606	0.801
Hs-CRP	1.032	1.015–1.049	≤0.001	1.002	0.997–1.047	0.091
Creatinine	0.993	0.981–1.005	0.245			
ALT	1.012	1.006–1.018	0.060			
AST	1.004	1.002–1.005	0.001	0.997	0.993–1.000	0.073
Total bilirubin	1.038	1.005–1.072	0.022	0.987	0.926–1.051	0.679
Fasting plasma glucose	1.018	1.030–1.135	0.002	0.908	0.862–0.956	0.363
Hematocrit	0.069	1.000–1.008	0.069			
BNP	1.001	1.000–1.001	0.073			
LVEF	0.872	0.847–0.899	≤0.001	0.908	0.862–0.956	≤0.001
LVEDD	1.174	1.121–1.231	≤0.001	1.136	1.016–1.271	0.025
LVESD	1.117	1.128–1.227	≤0.001	0.987	0.880–1.107	0.828
STEMI	2.983	1.583–5.623	0.001	0.772	0.319–1.886	0.565
Preinfarction angina	0.637	0.408–0.995	0.048	0.554	0.299–1.027	0.061
LAD (PCI)	1.995	1.252–3.180	0.004	0.888	0.164–4.818	0.891
RCA (PCI)	0.512	0.312–0.841	0.008	3.248	0.792–13.328	0.102
LAD (culprit)	2.042	1.293–3.225	0.002	1.232	0.222–6.827	0.811
RCA (culprit)	0.495	0.295–0.831	0.008	0.249	0.051–1.224	0.087
Single-vessel disease	1.586	1.020–2.465	0.040	2.037	1.067–3.888	0.031
TIMI-0 (before)	2.973	1.850–4.777	≤0.001	2.012	0.219–18.462	0.536
TIMI-3 (before)	0.334	0.206–0.541	≤0.001	0.993	0.100–8.688	0.952
IIb/IIIa inhibitor	1.777	1.124–2.809	0.014	1.192	0.616–2.305	0.603
*β*-Blocker	2.489	0.973–6.364	0.057			
Spironolactone	4.550	2.000–10.353	≤0.001	1.123	0.349–3.612	0.846

Hs-CRP: hypersensitive C-reactive protein; AST: aspartate aminotransferase; ALT: alanine aminotransferase; HDL-C: high-density lipoprotein cholesterol; BNP: B-type natriuretic peptide; LVEF: left ventricular ejection fraction; LVEDD: left ventricular end-diastolic diameter; LVESD: left ventricular end-systolic diameter; STEMI: ST-segment elevation myocardial infarction; LAD: left anterior descending artery; LCX: left circumflex artery; RCA: right coronary artery.

## Data Availability

The data that support the findings of this study are available from the corresponding author upon reasonable request.
